# Regional impacts of COVID-19 on carbon dioxide detected worldwide from space

**DOI:** 10.1126/sciadv.abf9415

**Published:** 2021-11-03

**Authors:** Brad Weir, David Crisp, Christopher W. O’Dell, Sourish Basu, Abhishek Chatterjee, Jana Kolassa, Tomohiro Oda, Steven Pawson, Benjamin Poulter, Zhen Zhang, Philippe Ciais, Steven J. Davis, Zhu Liu, Lesley E. Ott

**Affiliations:** 1 Universities Space Research Association, Columbia, MD, USA.; 2 Global Modeling and Assimilation Office, NASA Goddard Space Flight Center, Greenbelt, MD, USA.; 3 Jet Propulsion Laboratory, Pasadena, CA, USA.; 4 Cooperative Institute for Research in the Atmosphere, Colorado State University, Fort Collins, CO, USA.; 5 Earth System Science Interdisciplinary Center, University of Maryland, College Park, MD, USA.; 6 Science and Systems and Applications Incorporated, Lanham, MD, USA.; 7 The Earth from Space Institute (EfSI), Universities Space Research Association, 7178 Columbia Gateway Dr, Columbia, MD 21046, USA.; 8 Department of Atmospheric and Oceanic Science, University of Maryland, 4254 Stadium Dr, College Park, MD 20742, USA.; 9 Graduate School of Engineering, Osaka University, 2-1 Yamadaoka, Suita, Osaka 565-0871, Japan.; 10 Biospheric Sciences Laboratory, NASA Goddard Space Flight Center, Greenbelt, MD, USA.; 11 Department of Geographical Sciences, University of Maryland, College Park, MD, USA.; 12 Laboratoire des Sciences du Climat et de l'Environnement, Gif sur Yvette, France.; 13 Department of Earth System Science, University of California, Irvine, Irvine, CA, USA.; 14 Department of Earth System Science, Tsinghua University, Beijing, China.

## Abstract

Activity reductions in early 2020 due to the coronavirus disease 2019 pandemic led to unprecedented decreases in carbon dioxide (CO_2_) emissions. Despite their record size, the resulting atmospheric signals are smaller than and obscured by climate variability in atmospheric transport and biospheric fluxes, notably that related to the 2019–2020 Indian Ocean Dipole. Monitoring CO_2_ anomalies and distinguishing human and climatic causes thus remain a new frontier in Earth system science. We show that the impact of short-term regional changes in fossil fuel emissions on CO_2_ concentrations was observable from space. Starting in February and continuing through May, column CO_2_ over many of the world’s largest emitting regions was 0.14 to 0.62 parts per million less than expected in a pandemic-free scenario, consistent with reductions of 3 to 13% in annual global emissions. Current spaceborne technologies are therefore approaching levels of accuracy and precision needed to support climate mitigation strategies with future missions expected to meet those needs.

## Teaser

The regional impact of COVID-19 on atmospheric carbon dioxide is observable from space.

## INTRODUCTION

Reductions in human activity at the beginning of 2020 in response to the coronavirus disease 2019 (COVID-19) pandemic produced the largest short-term change in fossil fuel and cement carbon dioxide (CO_2_) emissions since the Industrial Revolution (*1*). Preliminary emission estimates for 2020 based on economic activity data suggest that, compared to 2019, daily global emissions decreased by as much as 15 to 20% in April (*2*). Accumulated from the start of the year, these reductions reached ~7.8% by June (*3*) and are expected to total ~4% (low estimate) to ~10% (high estimate) for the year, with the exact annual decrease depending on the intensity of the reduction during the lockdowns and the timing of the return of economic activity to pre-pandemic levels (*2*). Reductions in human activities were also indicated in satellite-observed changes in nighttime light intensity (*4*) and short-lived, combustion-related pollutants, e.g., nitrogen dioxide [NO_2_; (*5*–*7*)]. While activity-based estimates are consistent with reductions in satellite NO_2_ observations (*2*), the relationship of NO_2_ to CO_2_ emissions depends on combustion efficiency, which varies considerably across sectors and regions. Furthermore, CO_2_ emission estimates based on recent activity data, rather than the annual reported inventories typically used by “bottom-up” estimates, rely on different metrics and are thus subject to their own unique uncertainties. The two most well-known products (*2*, *3*), for example, intentionally produce estimates with non-negligible day-to-day variability and would benefit from independent verification and analysis, e.g., by comparison to energy data (*8*), their spatiotemporal disaggregations (*9*), and the estimates that follow.

For the past two decades, space agencies from around the world have planned and launched several satellite missions to observe vertical column average CO_2_ (XCO_2_) with a long-term goal of quantifying anthropogenic CO_2_ emissions and their trends. The current constellation includes Japan’s Greenhouse Gases Observing Satellite [GOSAT; (*10*)], launched in 2009; NASA’s Orbiting Carbon Observatory-2 [OCO-2; (*11*, *12*)] in 2014; Japan’s GOSAT-2 (*13*) in 2018; and NASA’s OCO-3, deployed in 2019 on the International Space Station (*14*). These missions were all designed as sounders that regularly sample the atmosphere at high precision, instead of mapping it in its entirety, with a strong focus on understanding the terrestrial biosphere. Future missions are expected to place an increasing focus on understanding anthropogenic emissions and improve coverage with greater swath widths and/or by sampling the atmosphere multiple times a day, e.g., NASA’s Geostationary Carbon Observatory [GeoCarb; (*15*)] positioned over the Americas and many other ongoing international efforts (*16*).

Developing a system that uses atmospheric CO_2_ observations to monitor changes in anthropogenic emissions remains a landmark achievement needed to support the implementation of international climate accords (*17*, *18*). Unlike NO_2_ observations, which display clear plumes with high concentrations over emitting areas, CO_2_ has a long lifetime in the atmosphere and is well mixed. Furthermore, in any given month, regional terrestrial biospheric fluxes have similar or greater magnitudes than fossil fuel emissions. This means that the CO_2_ signals caused by even large emission changes are confounded by those from long-range atmospheric transport and natural fluxes. To verify emission changes with atmospheric CO_2_ observations, the eventual goal is to sample the atmosphere as densely and frequently as possible above and downwind of emitting areas. This is not achievable with the current sparse surface network focused primarily on background CO_2_ but becomes increasingly possible with satellite observations. Below, we present our approach for monitoring changes in atmospheric CO_2_, analyze the observed changes in XCO_2_ in 2020, and demonstrate that our system can detect and quantify the impact of COVID-19 on XCO_2_, despite the difficulties noted in other studies (*19*–*21*). We conclude with a discussion of the scientific implications of those results.

## RESULTS

### Monitoring CO_2_ in near real time

**Fig. 1.**
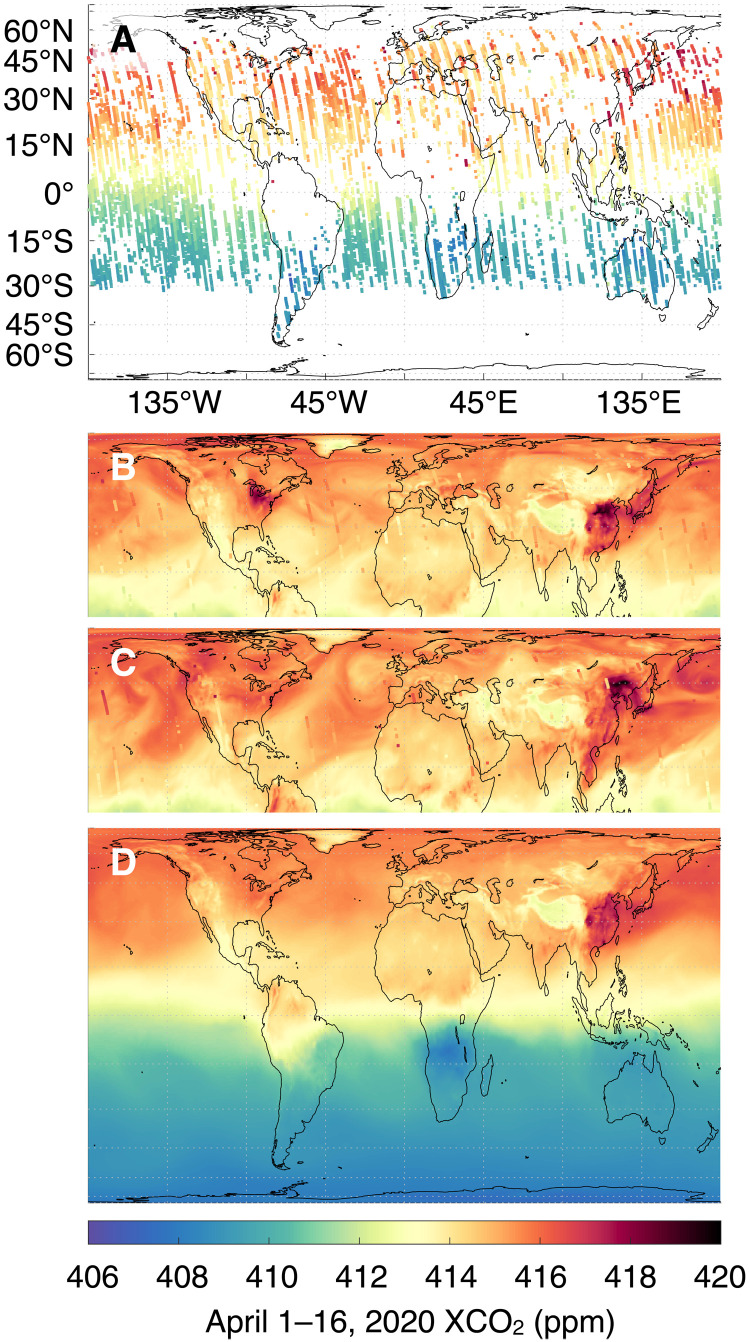
Snapshots of OCO-2 XCO_2_ soundings and assimilated GEOS/OCO-2 fields. (**A**) Sixteen days of OCO-2 XCO_2_ soundings on 1 to 16 April 2020. (**B** and **C**) Daily mean GEOS/OCO-2 XCO_2_ at the beginning, 1 April 2020 (B), and end, 16 April 2020 (C), of the 16-day period with 8 days of assimilated OCO-2 data overlaid on each. (**D**) The 16-day average of assimilated GEOS/OCO-2 XCO_2_ over the same period. DA combines satellite observations (A) with a weather-resolving atmospheric model (B and C) to form gridded, time-varying, 3D fields (fig. S1), from which averages (D) and uncertainties (Figs. 2 to 5) follow. Because it accounts for the several ppm changes in the Northern Hemisphere from (B) to (C) due to meteorological and submonthly flux variability, the assimilation system can detect and quantify the much smaller COVID-19 signal (see Materials and Methods and fig. S4). Monthly OCO-2 coverage and assimilated fits to data are depicted in figs. S2 and S3.

The Goddard Earth Observing System (GEOS)/OCO-2 atmospheric carbon monitoring system has several unique characteristics that enable it to capture and quantify the atmospheric signal due to COVID-19 related changes in activity [interactive visualizations available online at (*22*–*24*)]. First, it takes advantage of the unique coverage and precision of OCO-2 measurements. Mixed throughout the atmosphere, a 7% reduction in annual fossil fuel emissions represents just a 0.33–part per million (ppm) change (*25*, *26*) against the global marine boundary layer background concentration of 412.22 ppm in January 2020 (*27*), assuming that all other fluxes remain the same. While previous instruments have had insufficient coverage, accuracy, and/or precision to detect signals of this size, they remain within the nominal bounds of OCO-2 (*28*, *29*). Second, it uses coupled meteorology–carbon cycle components within GEOS (*30*) and data assimilation (DA) to infer three-dimensional (3D) gridded fields of CO_2_ for the entire OCO-2 data record, which can be averaged vertically and temporally as needed (see Materials and Methods, Fig. 1, and figs. S1 to S3). By using a transport model, our approach accounts for the year-to-year variability in CO_2_ due to differences in atmospheric circulation: Even with no change in surface fluxes, transport variability can cause several parts per million differences in XCO_2_ over the same area from 1 year to the next (*31*) and 1 day to the next (Fig. 1, B and C). This difference is especially relevant over North America, where passing weather systems cause sharp gradients across frontal boundaries (*32*). Analyses of XCO_2_ retrievals that do not account for transport variability (*20*, *21*, *33*) are therefore unlikely to capture year-to-year differences in emissions, especially given the sparse and infrequent sampling of OCO-2 over emitting areas. Our approach calculates anomalies against a simulated baseline surface flux scenario with the given year’s transport to account for known transport variability. Without this step, transport variability overwhelms the anomaly uncertainty (see Materials and Methods and fig. S4). Last, our system produces regular updates in near real time (NRT), taken here to mean a latency of less than a month, enabling the study of changes in the carbon cycle as they occur (*34*). Other common methods for inferring surface fluxes from atmospheric observations, e.g., flux inversion systems (*31*, *35*, *36*), typically trail the current date by several months or longer or are limited to a fixed period.

### Unprecedented CO_2_ anomalies in early 2020

**Fig. 2.**
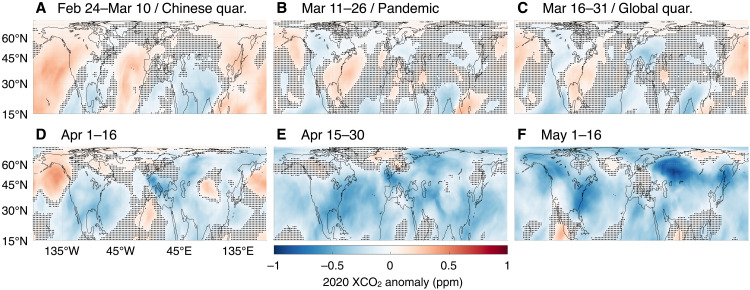
Spatial maps of GEOS/OCO-2 16-day moving average XCO_2_ anomalies over the Northern Hemisphere from late February to May 2020. Blue colors indicate decreases in XCO_2_ growth compared to a pandemic-free scenario (see Materials and Methods), while red colors indicate increases. Stippling indicates points where the signal is less than half an SD of the uncertainty. Before the COVID-19 pandemic anomalies in the Northern Hemisphere were a mixture of positive and negative with considerable uncertainty (**A**–**B**). Next, reductions surpassing 1 ppm, depicted in deep blue, developed over North America and Europe in mid-March through May (**C** to **F**) as COVID-19–related restrictions on activity were put in place. Afterward, in late May to early June, mixing by atmospheric transport, rebounds in emissions, and variability in the terrestrial biosphere diminish the magnitude and coherence of the COVID-19 signal (fig. S8).

**Fig. 3.**
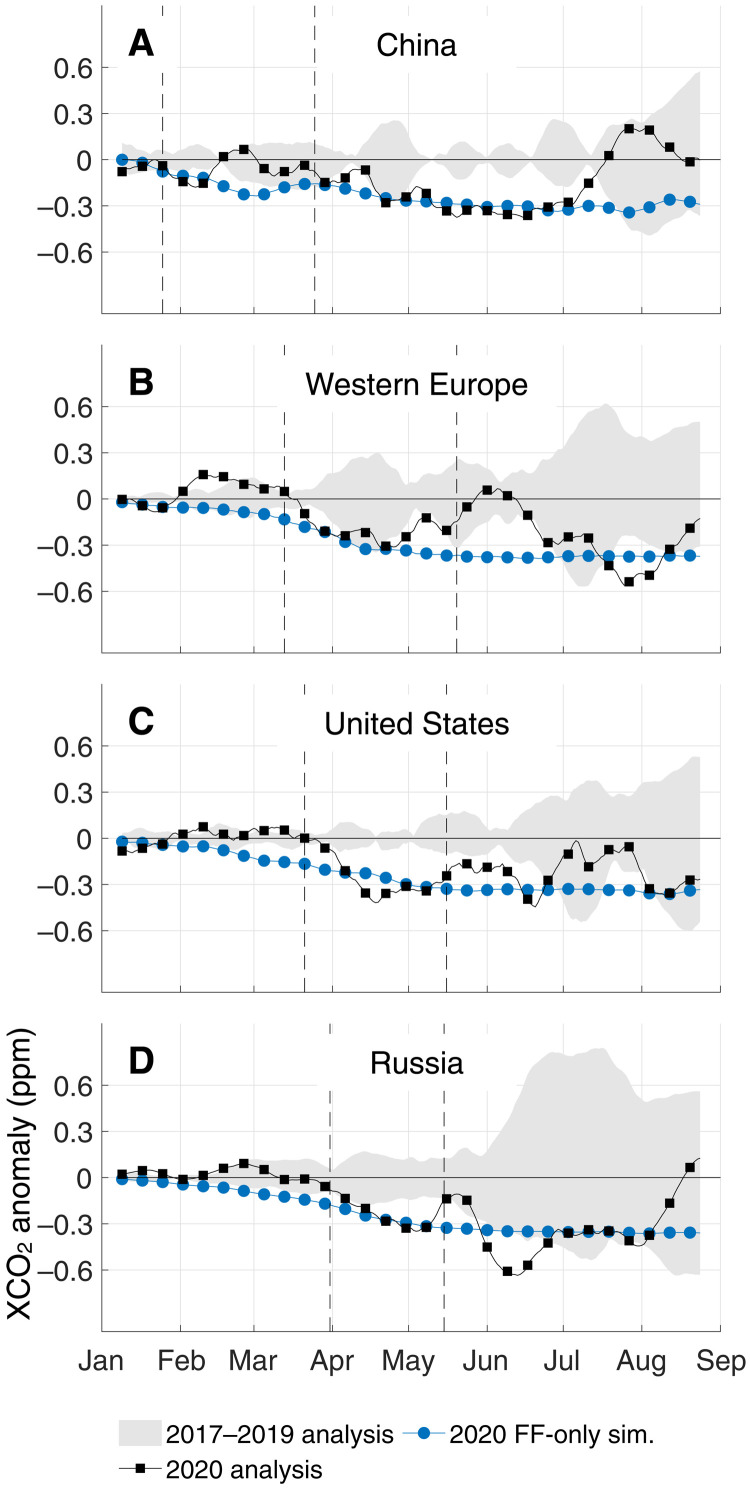
Time series of regional GEOS/OCO-2 16-day moving average XCO_2_ anomalies. (**A**) China, (**B**) Western Europe, (**C**) the United States, and (**D**) Russia. The solid black line with boxes indicates the 2020 anomaly, the gray shaded area indicates the spread of anomalies across the baseline years (2017–2019), and the blue circles indicate transport simulations for 2020 using activity-based fossil fuel (FF) emission estimates (*3*). Dashed lines mark a rough beginning and end (when appropriate) to confinements for each area and are provided in Table 1. For additional simulations and analysis including histograms of daily sounding counts, see fig. S8.

Over much of the Northern Hemisphere, home to most of the world’s largest economies and more than 95% of global total emissions, 16-day running means of XCO_2_ from the GEOS/OCO-2 analysis show consistent negative anomalies compared to a pandemic-free scenario (see Materials and Methods) beginning in February 2020 and continuing through May (Fig. 2). At the country/regional level, XCO_2_ anomalies show a steep initial decline coinciding with the implementation of activity restrictions and a subsequent leveling off with the relaxation of those measures (Fig. 3). This phasing corroborates the finding from activity-data indicators that emissions dropped precipitously during the initial confinement and then slowly recovered or plateaued (*1*–*3*): A simulation of the expected 2020 fossil fuel anomaly using the daily, activity-based estimates of (*3*) is depicted with blue circles in Fig. 3. Overall, our results and the bottom-up simulation agree about the magnitude of reductions in XCO_2_ growth at a country/regional level, with the analysis having slightly more temporal variability because it represents the anomaly from all fluxes, not just the fossil fuel component.

February–May 2020 anomalies over China, Europe, and the United States each exceeded the typical variability over the baseline period of 2017–2019 (see Materials and Methods): Peak 1σ uncertainties ranged from 0.14 to 0.32 ppm, while peak reductions in XCO_2_ growth reached 0.32 to 0.42 ppm (Table 1). By averaging those reductions and their uncertainties, we find a 1σ range of 0.14 to 0.62 ppm for the Northern Hemisphere. Assuming that the average reduction over the entire atmosphere is the same number at the end of the year, these estimates would produce 0.30 to 1.3 Pg C less of CO_2_ in the atmosphere (*25*, *26*), corresponding to a 3 to 13% reduction in the 10 Pg C global fossil fuel emission total estimated for 2019 (*37*). The conversion of ppm CO_2_ to Pg C used above (*25*) is only a rough indicator of emissions, especially because interhemispheric mixing takes more than a year to transport a signal from the Northern to Southern Hemisphere (*38*).

**Table 1.** GEOS/OCO-2 February–May regional reductions in XCO_2_ growth and associated uncertainties. Reductions and uncertainties are calculated from the data depicted in Fig. 3 as the peak 2020 reduction (solid boxes) and peak 1σ 2017–2019 uncertainty (gray shading) during February–May (see Materials and Methods). Start dates and end dates are taken from activity data (*3*). The average reduction over all four regions is 0.38 ppm, and average uncertainty is 0.24 ppm, giving a 1σ range of 0.14 to 0.62 ppm for the reduction over the Northern Hemisphere.

	**Peak reduction**	**Peak 1σ uncertainty**	**Start**	**End**
China	0.37 ppm	0.26 ppm	January 25	March 25
Western Europe	0.32 ppm	0.32 ppm	March 13	May 20
United States	0.42 ppm	0.14 ppm	March 21	May 16
Russia	0.41 ppm	0.22 ppm	March 31	May 15

The monitored changes in XCO_2_ over the Northern Hemisphere in February–May 2020 are primarily attributable to reductions in fossil fuel emissions for two reasons. First, late 2019 through 2020 saw neutral to weak La Niña conditions (*39*, *40*) of the El Niño–Southern Oscillation (ENSO). Globally, the annual growth rate of CO_2_ correlates well with a linear combination of total anthropogenic emissions and the Niño 3 or 3.4 ENSO index (*41*, *42*). The latter term, which serves as a proxy for biospheric variability, is small in 2020 (*39*, *40*), indicating a strong anthropogenic role in the growth rate anomaly. Regionally and monthly, ENSO remains a dominant driver of biospheric anomalies, but not without notable exceptions (*43*). Second, the months of February–May occur during a “shoulder” season in which net biospheric exchange is near its smallest (figs. S5 to S7), making it an ideal time to capture an anomaly driven by fossil fuel emissions. Transport simulations of 2020 anomalies from the Lund, Potsdam, Jena–Wald, Schnee und Landscaft [LPJ-wsl; (*44*, *45*)] and Catchment–Carbon and Nitrogen [Catchment-CN; (*46*)] terrestrial biosphere models (see Materials and Methods) also indicate that the biospheric anomalies in the Northern Hemisphere were relatively weak in February–May (fig. S7).

One notable disagreement between the GEOS/OCO-2 analysis and the bottom-up simulation is in the timing of the reduction over the United States. In the bottom-up simulation, reductions in XCO_2_ growth begin before activity restrictions as air with less CO_2_ is transported from China, across the North Pacific, and eventually to the United States, a process that takes several days. These reductions are not apparent in the monitoring system. Over China (Fig. 3A) and the North Pacific (Fig. 2), where we expect to see sustained reductions in XCO_2_, there is almost a complete rebound following the Lunar New Year. This is consistent with rebounds in NO_2_ observations from satellites (*5*) and in situ sensors (*7*). While another study (*6*) found a rebound in NO_2_ emissions following the Lunar New Year based on satellite observations, they did not find a complete recovery to pre-pandemic levels. There are several factors that could play a role in these discrepancies, each of which requires further investigation. In particular, uncertainties in Chinese emissions are greater than perhaps any other region (*47*, *48*), preventing us from making any strong conclusions about the magnitude of the recovery in their emissions. Nevertheless, these differences cannot be attributed to observational coverage or the DA system alone—an observing system simulation experiment (OSSE) that samples the simulated values at the time and place of OCO-2 soundings and assimilates the result is able to reproduce simulated signals (figs. S8 and S9)—nor can they be linked to anomalies in aerosol optical depth (fig. S10), which is a common cause of retrieval error. Last, companion simulations of biospheric anomalies suggest a small positive adjustment over the North Pacific and United States (fig. S7), although the difference is smaller than the within-model spread (indicated with stippling) and not great enough to account for the entire difference between the analysis and bottom-up simulation (yellow shading, fig. S8C). These results reinforce those of previous studies (*19*, *20*), which found it difficult to detect a COVID-19 signal over China using OCO-2 data.

**Fig. 4.**
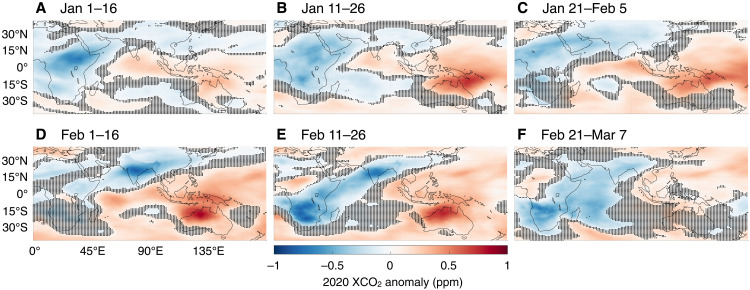
Identical to but Fig. 2 over the Indian ocean before the start of COVID-19–related confinements. In contrast to Fig. 2, the dominant signal shown here is from the carbon-climate teleconnection between the 2019–2020 Indian Ocean Dipole (IOD), the strongest on record, and terrestrial biospheric exchange. In January (**A** to **C**) and February (**D** to **F**) 2020, there was increased biospheric uptake over India and Africa (blue colors) due to greater-than-average precipitation in the preceding months, while there were increased respiration and biomass burning over Australia and Southeast Asia (red colors) due to greater-than-average temperatures.

**Fig. 5.**
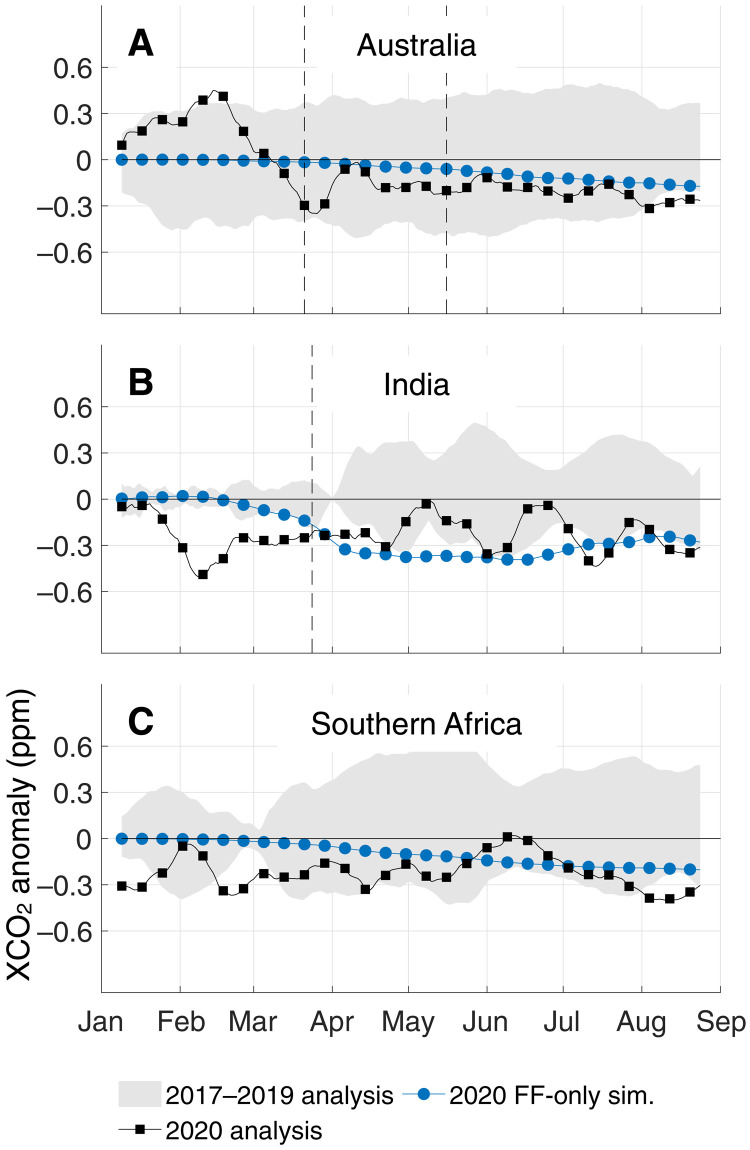
Identical to Fig. 3 but for different regions. (**A**) Australia, (**B**) India, and (**C**) southern Africa. The dominant signal is that of the IOD impact over India, but most of the anomalies are within the range of typical changes. As opposed to the Northern Hemisphere (Fig. 3), early in the calendar year is a time of substantial biospheric activity in the Tropics and Southern Hemisphere (figs. S5 to S7), complicating the interpretation of any anthropogenic variability. For corresponding histograms of daily sounding counts, see fig. S9.

While decreases in 2020 XCO_2_ growth due to COVID-19 were apparent in the Northern Hemisphere, the same cannot be said of the Tropics and Southern Hemisphere, where biospheric variability complicated the interpretation of any COVID-19 signal. Starting in 2019 and continuing through February 2020, GEOS/OCO-2 captured another notable change in XCO_2_, this time originating from the influence of a record-breaking climate anomaly on the terrestrial biosphere (Fig. 4). In 2020, well before their COVID-19–related restrictions, XCO_2_ growth dropped over India and sub-Saharan Africa and increased over Australia (Fig. 5). During this period, countries surrounding the Indian Ocean were experiencing the tail end of the 2019–2020 Indian Ocean Dipole (IOD) whose Dipole Mode Index was in the greatest positive phase in recorded history (*39*, *49*), setting monthly (October 2019) and 3-month average (September–November 2019) all-time highs. The impact of the IOD on the terrestrial biosphere and atmospheric circulation began in 2019, when both sub-Saharan Africa and India had wetter-than-usual boreal autumns; during the positive phase, cooler-than-normal sea surface conditions persist in the eastern Indian Ocean with warmer-than-normal conditions in the western tropical Indian Ocean (*50*–*52*). This East-West contrast in ocean conditions alters the wind, temperature, and rainfall patterns in the region, typically bringing mild temperatures and floods to sub-Saharan Africa and the Indian subcontinent (*53*) and high temperatures and droughts to East Asia and Australia (*54*), among other ecological and socioeconomic impacts. That increased rainfall over sub-Saharan Africa and the Indian subcontinent resulted in an extremely productive agricultural year and bumper crop harvests (*55*), while high temperature and drought conditions resulted in a record-setting fire season throughout Australia (*56*). The impact of these extremes on the carbon cycle persisted well into 2020, eventually falling off in early March (see Figs. 4 and 5 and the companion biospheric simulations in figs. S7 and S9).

## DISCUSSION

We found that satellite-monitored changes in early 2020 XCO_2_ due to of the COVID-19 pandemic were small (0.24 to 0.48 ppm), negative, and consistent with country-level activity data. The United States, Europe, and Asia each saw noticeable reductions in XCO_2_ growth coinciding with restrictions on activity and a return to typical growth as those restrictions were eased. Attribution of these signals to changes in anthropogenic emissions remains challenging: Interannual variability in transport and biospheric carbon-climate teleconnections both drive concentration changes many times greater than the record-setting changes in regional anthropogenic emissions due to COVID-19. For example, increased net vegetation growth in India and Africa and fires and respiration in Australia driven by the record-setting 2019–2020 IOD produced the greatest XCO_2_ anomalies of early 2020. The ability to detect fossil fuel CO_2_ emission changes in the midst of such climate variability is a milestone toward the long-term goal of monitoring future emissions, especially given the planned increase in space-based observing capability. Nevertheless, land and ocean flux variations related to ENSO and IOD, and their related uncertainties, continue to limit our ability to monitor and understand changes in anthropogenic emissions. Attribution of CO_2_ anomalies to individual surface flux components, and not their total, remains an active area of research with growing importance because of the societal need to reduce and monitor emissions. This effort will benefit in the future from improvements in terrestrial biospheric models; planned increases in space-based CO_2_ observations with a greater emphasis on fossil fuel emissions from NASA’s GeoCarb, Japan’s GOSAT constellation, and Europe’s CO_2_ Monitoring mission; colocation with other remote-sensing observations (e.g., NO_2_); and continued in situ measurement and scientific analysis of carbon isotopes, e.g., ^14^C in CO_2_ data (*57*).

## MATERIALS AND METHODS

### Data assimilation

The GEOS/OCO-2 atmospheric carbon monitoring system tracks changes in global atmospheric CO_2_ every 3 hours by ingesting OCO-2 Build 10 XCO_2_ full-physics retrievals (*58*, *59*) into GEOS using a statistical technique commonly referred to as DA and/or state estimation (*60*, *61*). It has been previously documented for an earlier version of OCO-2 data (*12*) and available to the public on the NASA/ESA/JAXA trilateral Earth Observing Dashboard (*22*) and NASA COVID-19 Dashboard (*23*) since July 2019.

DA synthesizes simulations and observations, adjusting the state of atmospheric constituents such as CO_2_ to reflect observed values, thus gap-filling the observations when and where they are unavailable. These features are particularly appealing given the narrow, 10-km-wide swath and 16-day repeat time of OCO-2 (Fig. 1 and fig. S2). Under sufficiently general assumptions, DA can be considered a machine learning (ML) method. However, compared to interpolation, Kriging, and most other ML approaches, DA has the advantage that it makes estimates based on our collective scientific understanding of Earth’s carbon cycle, as encapsulated within GEOS, rather than relying on functional relationships that rarely hold in nature. The value of relying on forecasted fields instead of functional relationships in data analysis has been understood in the numerical weather prediction community since at least 1954 (*62*), even before E. Lorenz’s seminal work (*63*), yet receives less attention in other disciplines. Figure 1 demonstrates the impact of DA on OCO-2 coverage for April 2020. Before assimilation (Fig. 1, top), there are notable patches of missing data where either clouds (e.g., the Amazon), aerosols (China), and high solar zenith angles (the poles) prevent reliable measurements. Assimilation produces 3D CO_2_ fields with global coverage that are updated every 3 hours (Fig. 1, middle, and fig. S1). Values in missing areas are inferred from nearby observational data and model relationships. The 16-day running means (bottom) and monthly means analyzed here follow from simple averaging of the assimilated CO_2_ fields.

GEOS/OCO-2 uses the GEOS Constituent Data Assimilation System (CoDAS), a high-performance computing implementation of Gridpoint Statistical Interpolation [GSI; (*64*)], a technique for finding the analyzed state that minimizes the 3D variational (3D-Var) cost function formulation of the differences between observed and simulated values. GEOS CoDAS ingests column retrievals of trace gas abundances, accounting for both their vertical sensitivity (i.e., averaging kernel) and a priori assumptions. While current versions of GSI include the ability to use 4D variational (4D-Var) and hybrid ensemble-variational formulations (*65*), this application relies on the simpler 3D-Var technique. In GEOS CoDAS, the atmospheric circulation is constrained by the millions of remote-sensing and in situ observations every hour included in the Modern Era Retrospective analysis for Research and Application, version 2 [MERRA-2; (*66*)]. This accurate representation of transport patterns at fine spatial resolutions is critical for interpreting measured variations that reflect a combination of nearby and distant surface fluxes due to the long lifetime of CO_2_ and helps us reproduce atmospheric observations with high fidelity in the marine boundary layer (*34*) and over North America, where there is a wealth of data, e.g., airborne in situ measurements from NASA’s Atmospheric Carbon and Transport–America (ACT-America) campaign (*67*). Extensive evaluation against these data, which are withheld from the assimilation, makes us confident in the ability of GEOS/OCO-2 to estimate regional signals with small magnitudes. In other applications, GEOS CoDAS has been used to analyze multidecadal trends of stratospheric ozone (*68*) and the anomalously small ozone hole of 2019 (*69*).

All GEOS CoDAS runs here use a similar methodology and setup to that described in (*69*) and the references therein. The horizontal grid has a nominal resolution of 50 km, and there are 72 vertical levels starting from the surface, where they follow the terrain, and extending up to 0.01 hPa, where they follow fixed pressure values. The assimilation system processes observations in 6-hour intervals. At the beginning of each interval, it uses the GEOS model to simulate a 6-hour forecast/background and saves output every 3 hours for the purpose of time interpolation. It then solves for the minimum value of the cost function

$$J(x)=\frac{1}{2}{\left(x-x_{b}\right)}^{T}B^{-1}(x-x_{b})+\frac{1}{2}{\left(y-\mathrm{Hx}\right)}^{T}R^{-1}(y-\mathrm{Hx})$$

where *x* is the state vector of trace gas values at all grid points, *y* is the observation vector, *H* is the observation operator, *B* is the background error covariance matrix, and *R* is the observation error covariance matrix. This formulation abuses notation slightly as the GSI 3D-Var formulation assumes that the same increment *x* − *x_b_* is constant throughout the 6-hour interval, while using 3-hourly temporal interpolation for the evaluation of *Hx*. GEOS/OCO-2 uses a homogeneous, horizontally isotropic background error covariance *B* whose diagonal is 0.15 ppm everywhere, with a nominal horizontal error correlation length of 500 km and vertical error correlation length proportional to the vertical correlation length of the given tracer. The observation error covariance *R* uses the reported retrieval error variances scaled by a factor of 0.85^2^ as its diagonal and has no cross-sounding correlations. While these crude error models could be improved, a posteriori diagnostics and evaluation against independent data indicate that the system is sufficiently well tuned. As an additional level of quality control, we do not assimilate retrievals that are over snow and ice, have a glint angle greater than 80°, or are in a swath with less than four footprints. Soundings with a reported uncertainty less than 0.001 ppm are also flagged and not assimilated. Cross-track variability of XCO_2_, accounting for the retrieval mode and surface type, is included in the retrieval errors by geometrically averaging it with the reported values. The final step for each interval is to rerun the 6-hour forecast with the optimal increment x^*^ − *x_b_*, where x^*^ minimizes the cost function *J*, applied as a forcing in the same manner as for the meteorological variables (*65*).

Data processing is divided into six separate streams covering 2015–2020. Each stream begins on 31 October of the previous year to allow some equilibration of the analysis before the period of interest beginning on 1 January. Differences between overlapping streams are less than 10% of the magnitude of the anomalies analyzed here and thus can be safely ignored. The results presented here use no CO_2_ data other than OCO-2 observations in the present year, here 2020. In previous years, it uses a single number, the atmospheric growth rate, to set the global flux budget as described below.

A unique feature of GEOS/OCO-2 is its ability to process data in NRT, as retrievals become available to assimilate. This is accomplished primarily through the use of a surface flux collection, the Low-order Flux Inversion [LoFI; (*34*)] with distinct modes for retrospective and NRT simulations. In retrospective simulations, the system uses surface fluxes informed by several remote-sensing datasets that include fire radiative power, nighttime lights, and vegetation properties such as leaf area index (*30*) and atmospheric growth rate estimates derived from surface observations. In NRT, before many of these datasets become available, LoFI uses fluxes and a projected atmospheric growth rate based on data from previous years and the current ENSO phase (*41*, *42*). This dual capability ensures a strong, multiplatform data constraint on XCO_2_ on previous years for computing anomalies, while the products for the current year highlight areas where land, ocean, and fossil fuel fluxes deviate from expectations. For fossil fuel emissions, we use the 2018 version of the Open-source Data Inventory for Anthropogenic CO_2_ [ODIAC; (*70*)], which estimates emissions by tracking fossil fuel consumption (i.e., barrels of oil and tons of coal) and cement production (*9*) and ends in 2018. For 2019, we rescale the 2018 monthly gridded maps to match the Global Carbon Project 2019 global emission estimate (*37*), and for 2020, we simply repeat 2019 emissions.

### Anomalies and pandemic-free baseline atmospheric CO_2_ fields

Even after constructing gap-filled XCO_2_ maps, defining 2020 anomalies for CO_2_ is more challenging than for most other species. For NO_2_, which is short-lived, simply subtracting a multiyear climatological mean from the 2020 values is often sufficient for highlighting recent emission changes (*5*), although recent research suggests that meteorological variations can play an important role in the interpretation of NO_2_ changes (*7*, *71*). For CO_2_ and other long-lived species, anomalies calculated against a climatological baseline reveal a strong imprint of circulation anomalies, which can have a greater impact than and obscure the spatial signature of emission changes.

To minimize the circulation influence, at the beginning of each year, we start a companion GEOS simulation that is identical to the analyzed product, except that OCO-2 data are not assimilated. By subtracting the simulated anomaly from the analysis anomaly, we isolate the flux-driven signal observed by OCO-2 from the transport variability–driven signal. We refer to this difference as the “analysis correction.” The pandemic-free, baseline scenario is then the average of all analysis corrections for 2017–2019 plus the GEOS simulation for 2020. This represents 2020 transport while applying the mean analysis corrections due to assimilating OCO-2. Subtracting the pandemic-free field from the 2020 analysis then gives the flux-driven GEOS/OCO-2 anomaly depicted in Figs. 2 to 5 and in the supplementary figures. Figure S4 depicts the difference between this anomaly calculation, which we call “transport aware,” and an anomaly calculation that uses a simple climatology of previous years as a baseline. By not accounting for year-to-year transport variability, the latter has a much greater SD across years, as seen in the increased stippling in fig. S4.

We omit 2015 and 2016 from our baseline years because they contain one of the strongest ENSOs on record and are not representative of 2020, which was neutral in the first 3 months of 2020 and transitioned to a moderate La Niña in April 2020 (*38*, *39*). Strong ENSO signals produce substantial interannual variability in carbon fluxes over ocean and land (*35*, *72*) as well as atmospheric circulation patterns (*73*). Figures S11 and S12 add the 2015 and 2016 anomalies onto the plots from Figs. 3 and 5. The ENSO years (red) are clear outliers, supporting their exclusion from the analysis.

### Uncertainty quantification

As an indicator of uncertainty, we use the range of analysis corrections for individual years in 2017–2019 depicted as the gray shading in Figs. 3 and 5. From the ranges, we calculate the 2σ uncertainty as half the minimum-to-maximum range of the gray shaded area, corresponding to an assumption that the 2017–2019 range represents about 95% of year-to-year variability in neutral ENSO conditions. The uncertainty ranges reported in Table 1 are consistent with evaluations of GEOS/OCO-2 against independent surface in situ and remote-sensing observations and a posteriori tests of the statistical consistency of the DA system (see the Supplementary Materials). They are smaller than but the same order as the errors reported by the analyses in several previous studies (*19*, *20*, *74*–*76*). This is to be expected as our uncertainty estimate does not include persistent biases, while those estimates do. They also coincide with a 0.15 ppm SD of the analysis error uncertainty for the GEOS/OCO-2 fields calculated from an a posteriori diagnostic (*77*).

### Separating the COVID-19 atmospheric CO_2_ signal from natural variability

To help separate anthropogenic from natural variability, we perform two supplementary GEOS CO_2_ simulations. The first transports the difference in 2020 and 2019 emissions from the daily activity-based fossil fuel estimates (*3*) through the atmosphere using the same settings as the GEOS/OCO-2 assimilation run (monthly global maps in fig. S7). For these simulations, daily country-level emission totals for 2019 and 2020 are spatially downscaled using 2015 monthly EDGAR v5.0 sector totals (*78*) for power generation, ground transportation, industry, aviation, residential energy usage, and international shipping. The second simulation aims to represent the difference in 2020 biospheric flux by transporting the difference between 2020 and the 2017–2019 average calculated using the LPJ-wsl dynamic global vegetation model (figs. S5 to S7). While LPJ-wsl is a different model of the terrestrial biosphere than we use for our prior fluxes, it is useful as a prognostic, independent method of identifying regional biospheric anomalies and has been demonstrated to realistically reproduce interannual variations in global net flux (*37*). For consistency, we apply the same MERRA-2 meteorological data used to force our transport simulations and as inputs to LPJ-wsl (*45*) and Catchment-CN (*46*).
