# Supplementary material for: Regional impacts of COVID-19 on carbon dioxide detected worldwide from space
Source: Sci Adv. 2021 Nov 3;7(45):eabf9415. doi: 10.1126/sciadv.abf9415 (PMC8565902; doi:10.1126/sciadv.abf9415)
Supplement: Supplementary file 1 — Figs. S1 to S12 [file sciadv.abf9415_sm.pdf]

Science Advances
AAAS

Supplementary Materials for

Regional impacts of COVID-19 on carbon dioxide detected worldwide from space

Brad Weir*, David Crisp, Christopher W. O'Dell, Sourish Basu, Abhishek Chatterjee, Jana Kolassa, Tomohiro Oda, Steven Pawson, Benjamin Poulter, Zhen Zhang, Philippe Ciais, Steven J. Davis, Zhu Liu, Lesley E. Ott

*Corresponding author. Email: brad.weir@nasa.gov

Published 3 November 2021, *Sci. Adv.* **7**, eabf9415 (2021)
DOI: 10.1126/sciadv.abf9415

This PDF file includes:

Figs. S1 to S12

Supplementary Materials

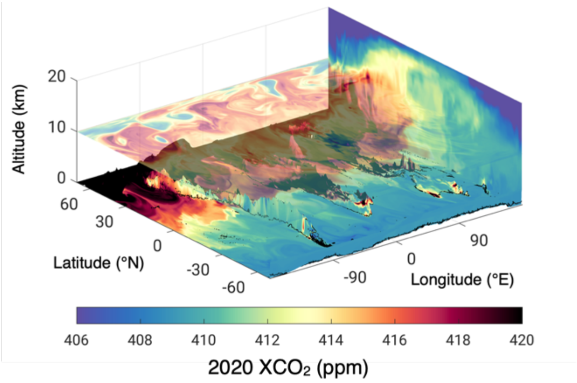

Fig. S1. Transects of instantaneous GEOS/OCO-2 CO_2 on April 9, 2020 at 00:00 UTC at the surface (bottom), 10 km above sea level (top, transparent), and along the International Dateline (right). By reproducing the global, high-resolution, vertical and temporal variability of CO_2, the assimilation system can synthesize heterogenous data types across drastically different scales, e.g., satellite retrievals and in situ measurements from surface stations and aircraft.

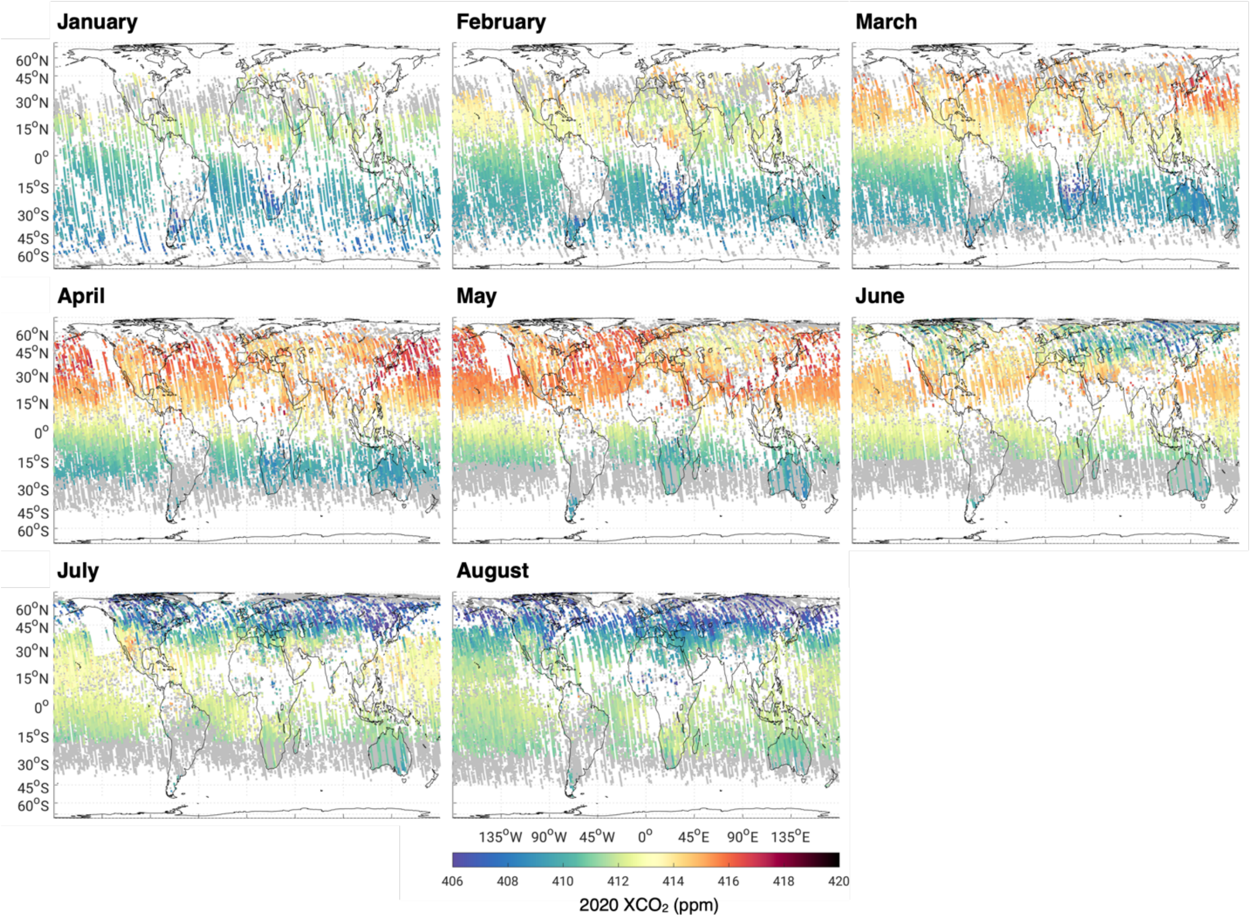

Fig. S2. Monthly maps of OCO-2 B10 XCO_2 retrieval coverage. The OCO-2 satellite flies in a sun-synchronous, low-Earth orbit with a local overpass time of 1:30 PM in NASA's Afternoon Train (A-Train) formation. Soundings flagged by our additional level of quality control are depicted in gray (see Materials and Methods). Its most significant effect is on coverage at high solar zenith angles over the ocean and Southern Hemisphere land.

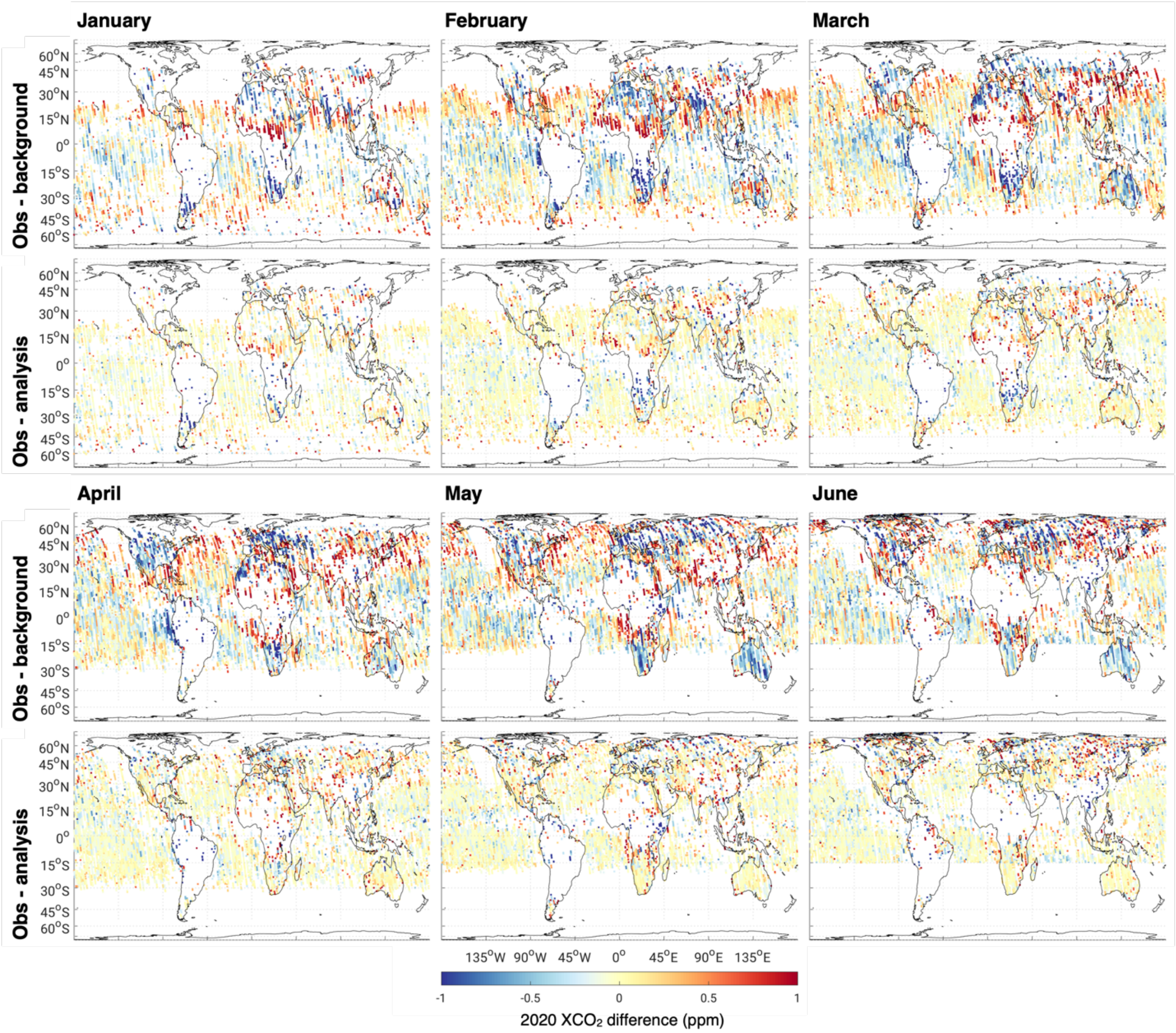

Fig. S3 (continued below ...). Monthly maps of OCO-2 minus GEOS/OCO-2 background (before assimilation; top rows) and analysis (after assimilation; bottom rows) XCO_2. Assimilation clearly improves the fits to the assimilated data, as intended. Differences after assimilation have O(0.1 ppm) magnitudes, further supporting the uncertainty quantification in the paper. Only assimilated soundings, which pass the additional quality filters (see Materials and Methods) and are thus depicted in color in Fig. S2, are shown here. A final layer of quality control, which discards data whose absolute difference with the model exceeds 3.5 standard deviations of the observation error, accounts for sporadic data points depicted in Fig. S2 and missing here.

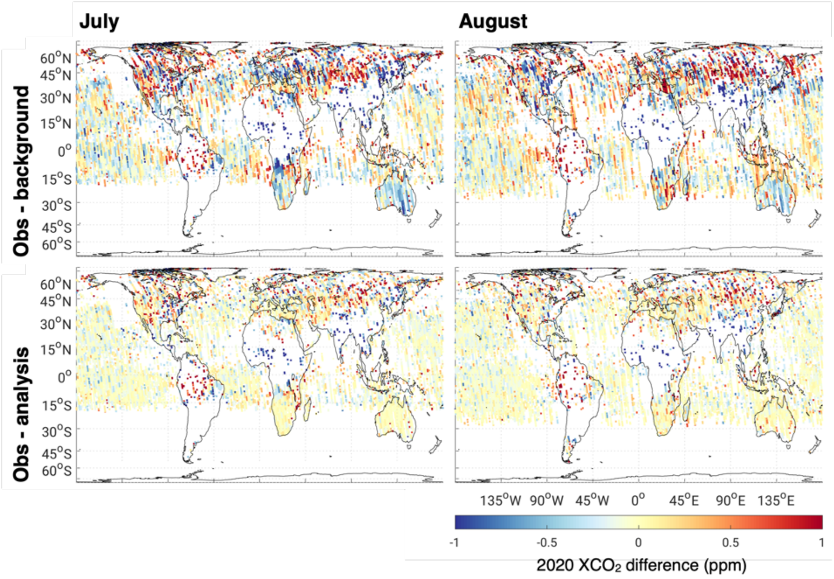

Fig. S3 (... continued from above).

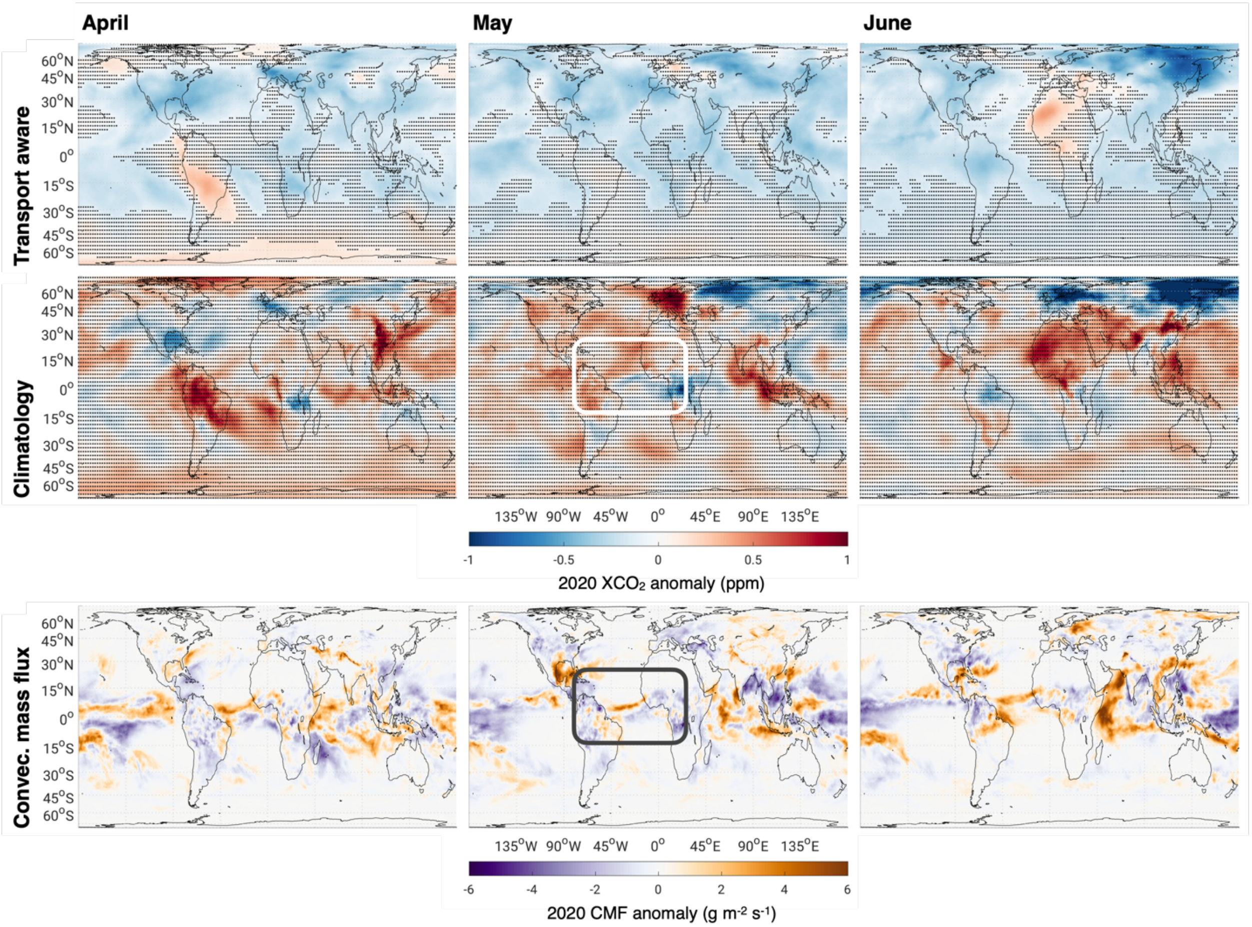

Fig. S4. Anomalies of GEOS/OCO-2 monthly average XCO_2 during early 2020 computed with two different pandemic-free baseline scenarios along with anomalies in convective mass flux from MERRA-2. The first row, used in the text, computes a baseline that accounts for year-to-year variability in atmospheric transport by subtracting out simulated values. The second row computes a baseline that is the climatology of previous years with a constant offset and makes no adjustment for inter-annual variability in transport. Stippling in both indicates when the signal is less than half a standard deviation. By not accounting for transport, the latter anomaly estimate produces a signal with far greater uncertainty, so much so that every point is stippled. In addition, the large positive/negative anomaly over the tropical Atlantic in May 2020 (inset box) is a direct result of year-to-year changes in convective mass flux (bottom row) and not due to a surface flux anomaly, which we hope to detect. Similar plots for anomalies in sea-level pressure and convective mass flux for each month are given in Fig. S12.

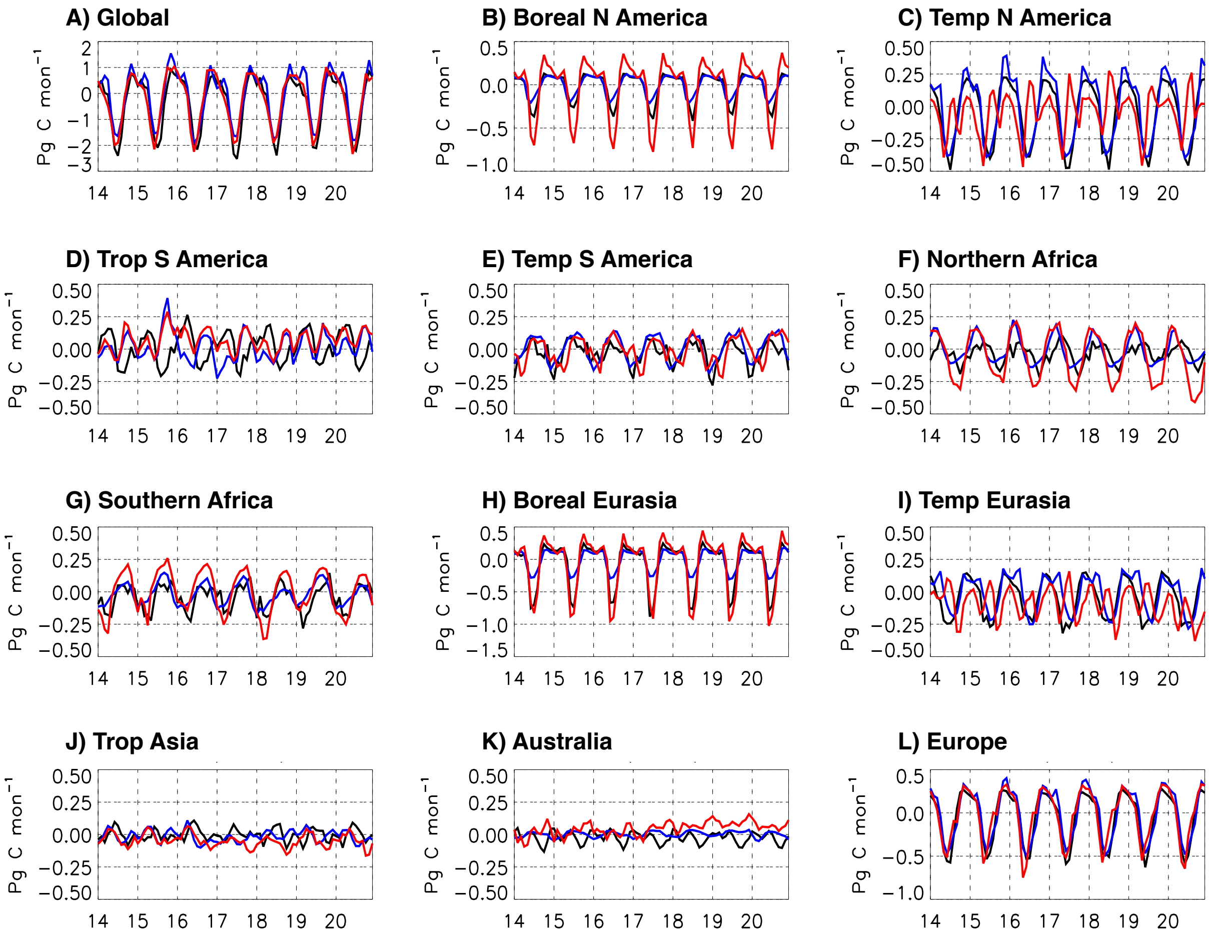

Fig. S5. NBE from our prior surface fluxes (LoFI, black), the LPJ-wsl model (red), and the Catchment-CN model (blue) used for the biospheric anomaly simulation. While the models reproduce roughly consistent global seasonal cycles (A), LPJ-wsl has a seasonal cycle that is about twice as great as LoFI in Boreal North America, North Africa, and South Africa, and Catchment-CN has a seasonal cycle about twice as small as LoFI and LPJ-wsl in Boreal Eurasia. These differences suggest a COVID-19 signal in XCO_2 is most likely to be detected in February–May before the greatest uncertainties among the models occur.

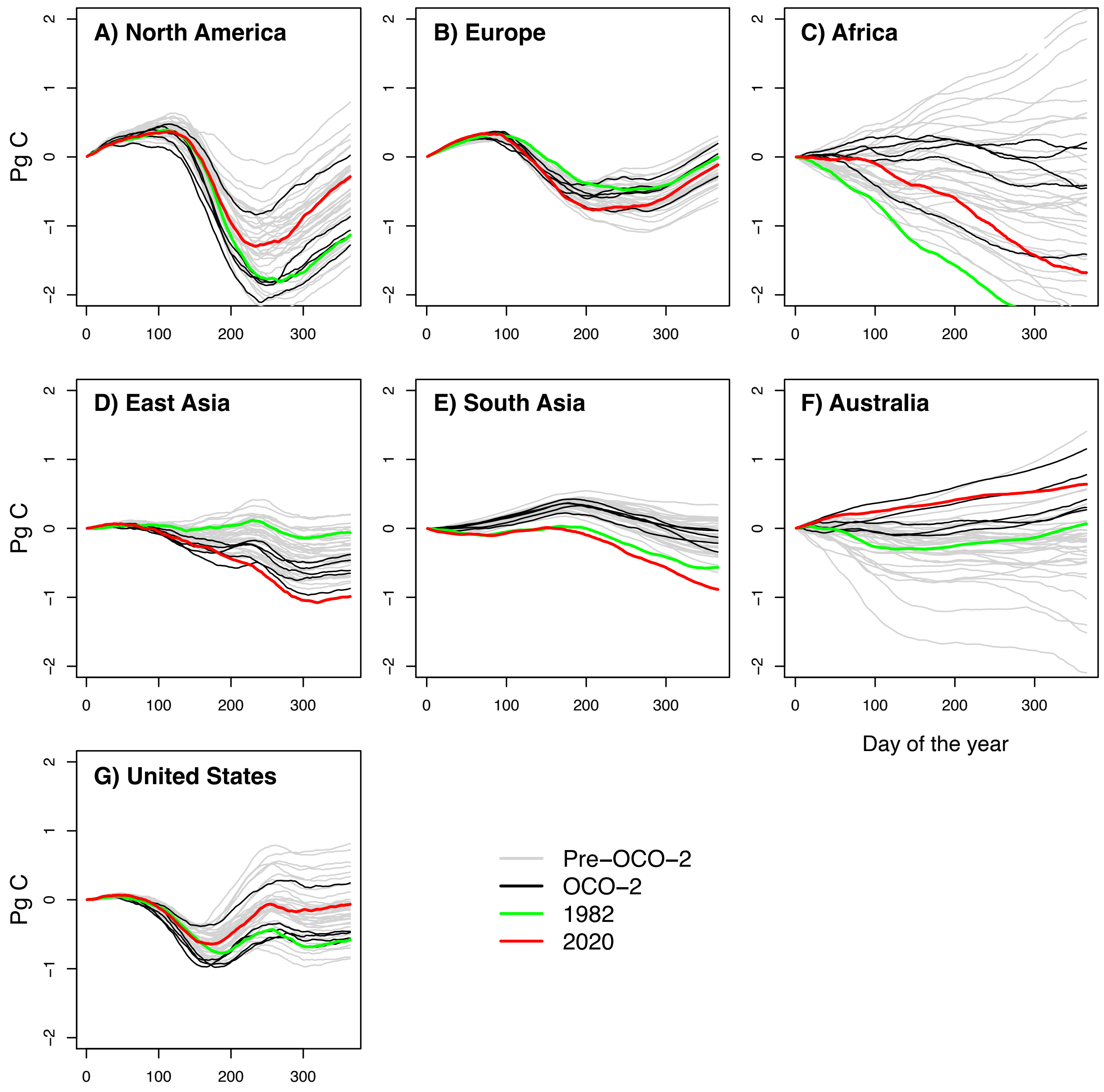

Fig. S6. Cumulative NBE anomalies from LPJ-wsl terrestrial biospheric model over different countries/regions. Each line represents a different year with years before the OCO-2 launch (2014) drawn in gray, years after the OCO-2 launch drawn in black, 1982 drawn in green (most recent IOD of comparable magnitude), and 2020 drawn in red. The year 2020 is a clear outlier in South Asia and Oceania/Australia, indicating strong biospheric anomalies. Over North America, Europe, and East Asia (viz., China), 2020 is a typical year for the biosphere, if not more of a source (positive values) of carbon to the atmosphere. The rapid increase in inter-annual variability in the Northern Hemisphere around the end of May (day 150), shows that a fossil fuel anomaly is best observed during its winter and spring.

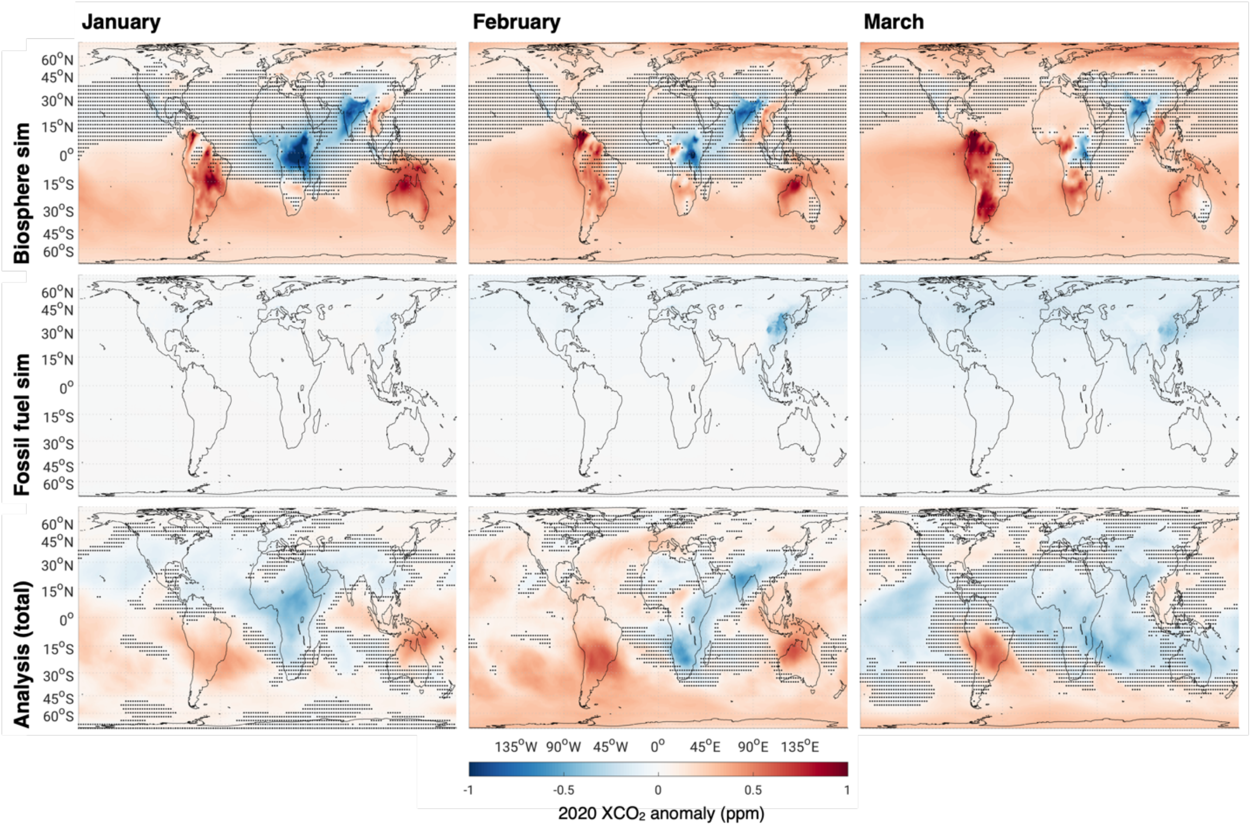

Fig. S7 (continued below ...). Monthly maps of XCO_2 in ppm from biospheric anomaly simulations (top), fossil fuel anomaly simulation (middle), and GEOS/OCO-2 analysis anomaly (bottom) with stippling indicating signals smaller than half a standard deviation. In January through February, the GEOS/OCO-2 anomaly captures the IOD signal over India, Australia, and Africa, showing remarkable agreement with the spatial patterns from the biospheric simulations. The amplitudes of those patterns are nevertheless quite different, with LPJ-wsl estimating a seasonal amplitude more than twice that of LoFI in some regions (Fig. S5).

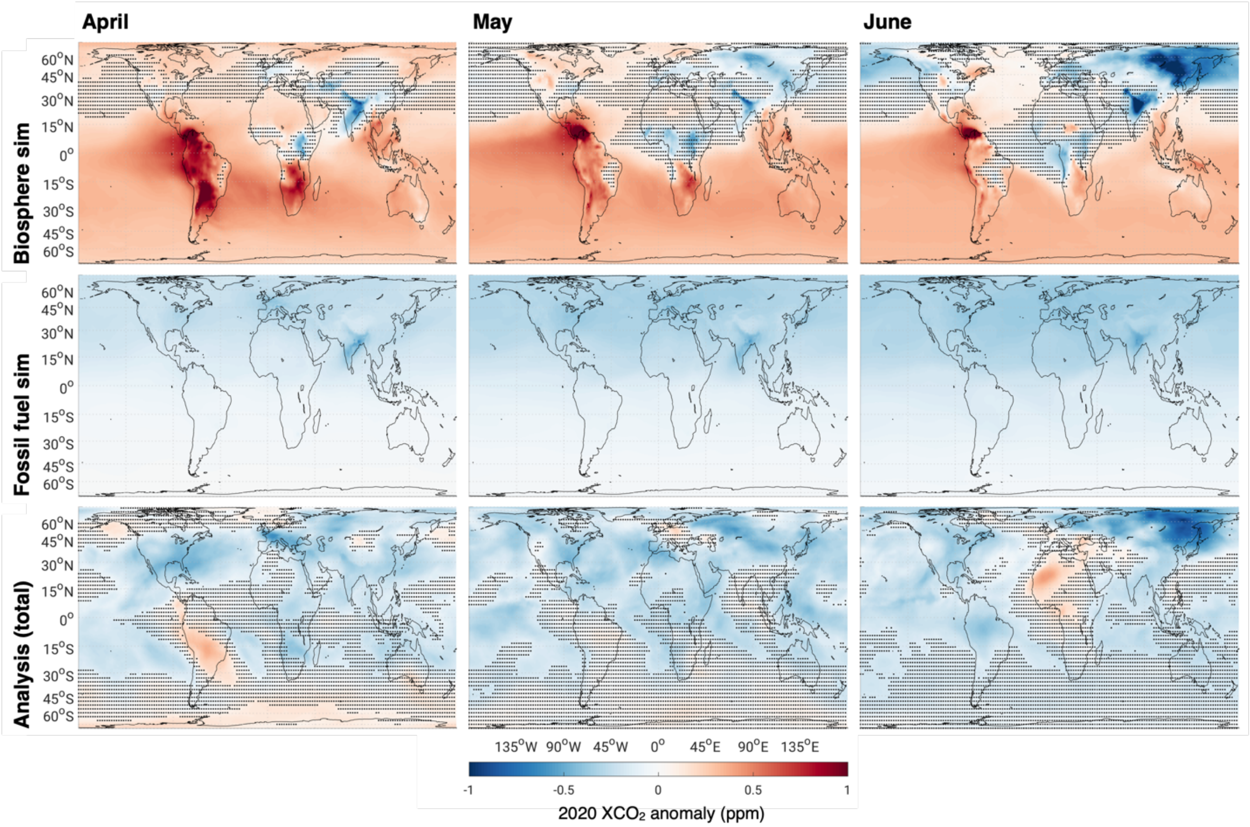

Fig. S7 (... continued from above and below ...). In February–May, the simulated biospheric variability over the United States, Europe, Russia, and China simulated remains relatively small and, if anything, net positive, supporting the attribution of the analysis anomalies to fossil fuel emissions. In June, biospheric variability begins to dominate in the Northern Hemisphere, complicating the interpretation of any anthropogenic signals (cf. the growth into June and July of the gray shaded regions in Fig. 3), e.g., the large negative analysis anomaly over Siberia in June (top right) is most likely due to the biosphere (middle right).

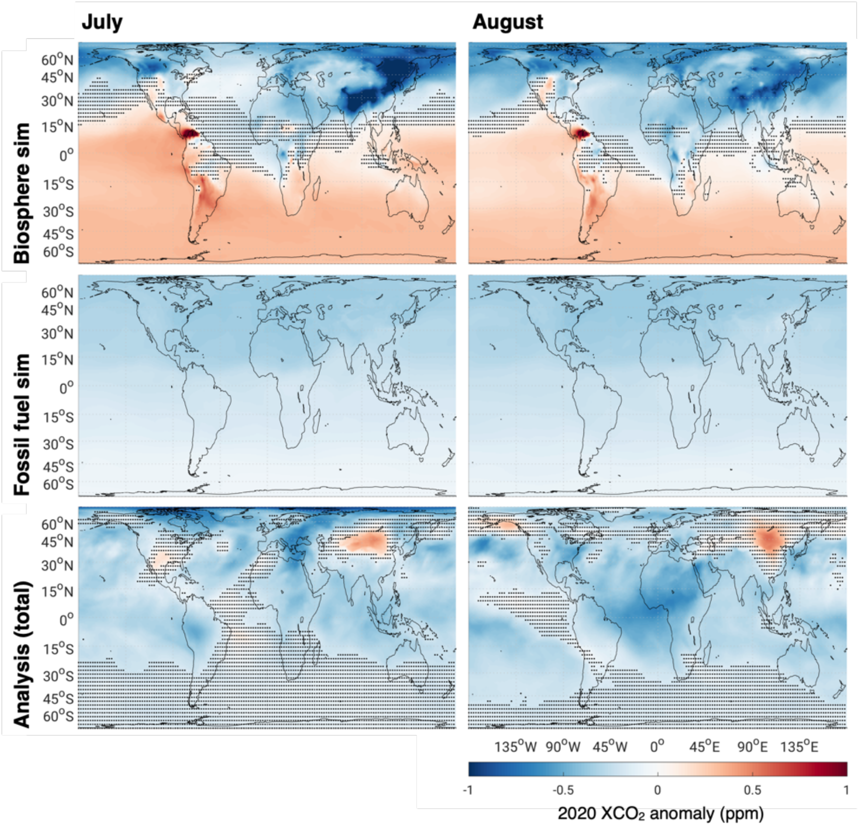

Fig. S7 (... continued from above).

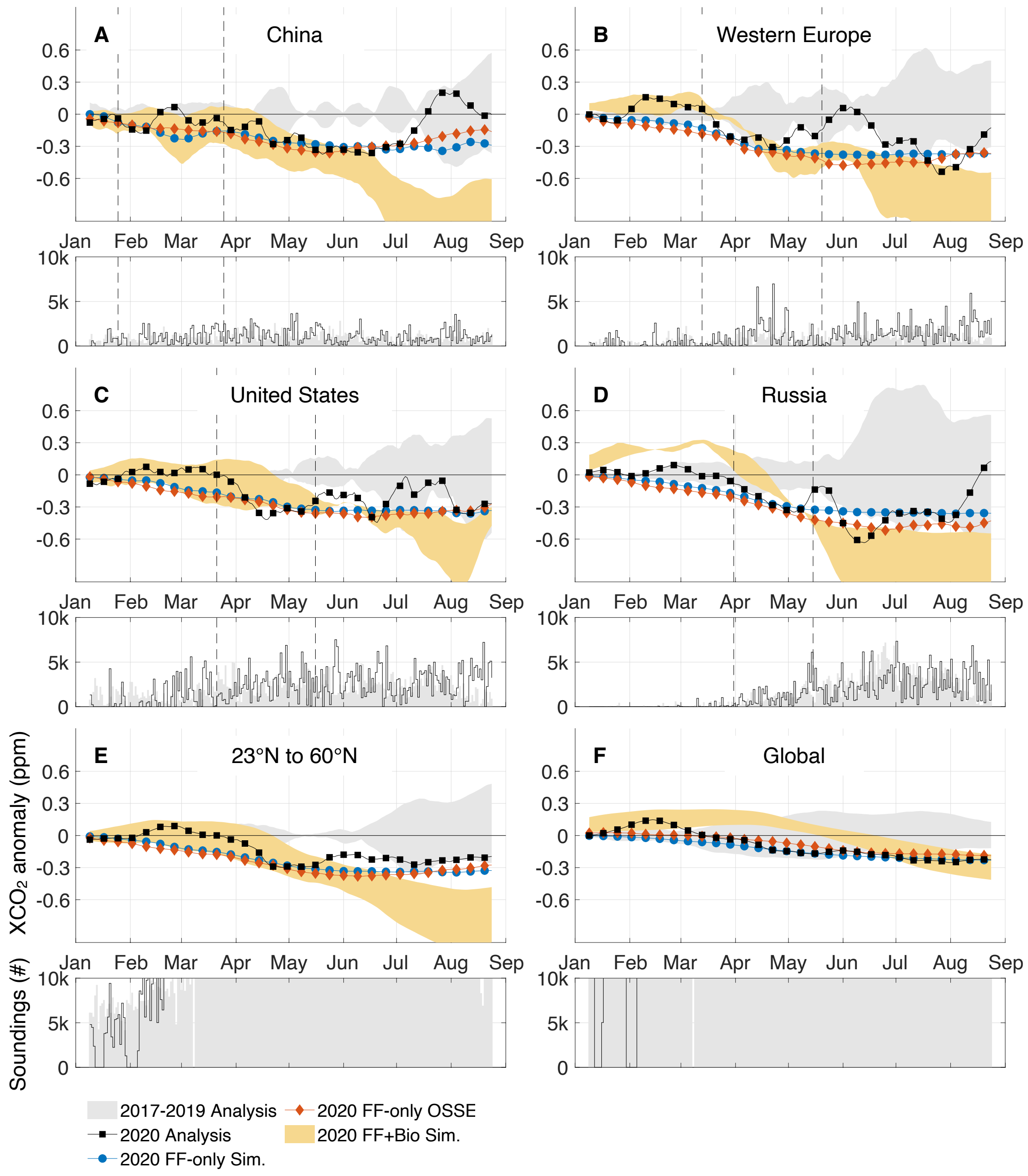

Fig. S8. Identical to Fig. 3 with fossil fuel OSSE results (red diamonds), fossil fuel plus biosphere anomaly simulations (yellow shading), and daily soundings (lower panel) for 2020 (black line) and the 2017–2019 average (gray shading) included. The proximity of the fossil fuel OSSE results (red diamonds) to the simulated values (blue circles) demonstrates that the data coverage and assimilation system can reliably capture the signal of activity-based emissions reduction estimates. The distance from the blue circles to the yellow shaded area indicates the magnitude and spread of the simulated biospheric anomalies, and the distance from the black squares to the yellow shaded area the consistency of the analysis anomaly and the simulated anomalies. The agreement of the blue circles, black boxes, and yellow shaded area in the Northern Hemisphere during February–May 2020 supports the conclusion that the assimilation system captured decreases in fossil fuels emissions due to COVID-19.

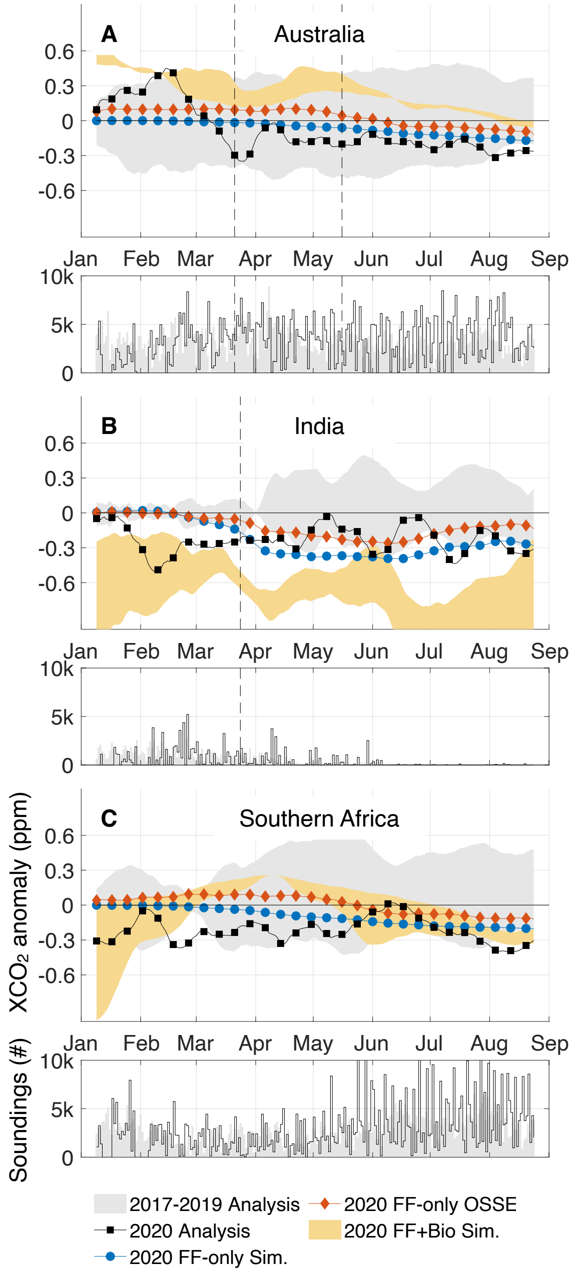

Fig. S9. Identical to Fig. 5 with OSSE results (red diamonds), fossil fuel plus biosphere anomaly simulations (yellow shading), and daily soundings (lower panel) for 2020 (black boxes) and the 2017–2019 average (gray shading). While the OSSE results (red diamonds) reproduce the general features of the simulated values (blue circles), the analysis has greater inter-annual variability than in the Northern Hemisphere (compare gray shading in Fig. S8) consistent with a greater biospheric anomaly (distance from blue circles to yellow shaded area). See also the difference between the biospheric (top column) and fossil fuel only (middle column) anomaly maps in the Tropics and Southern Hemisphere in Fig S7.

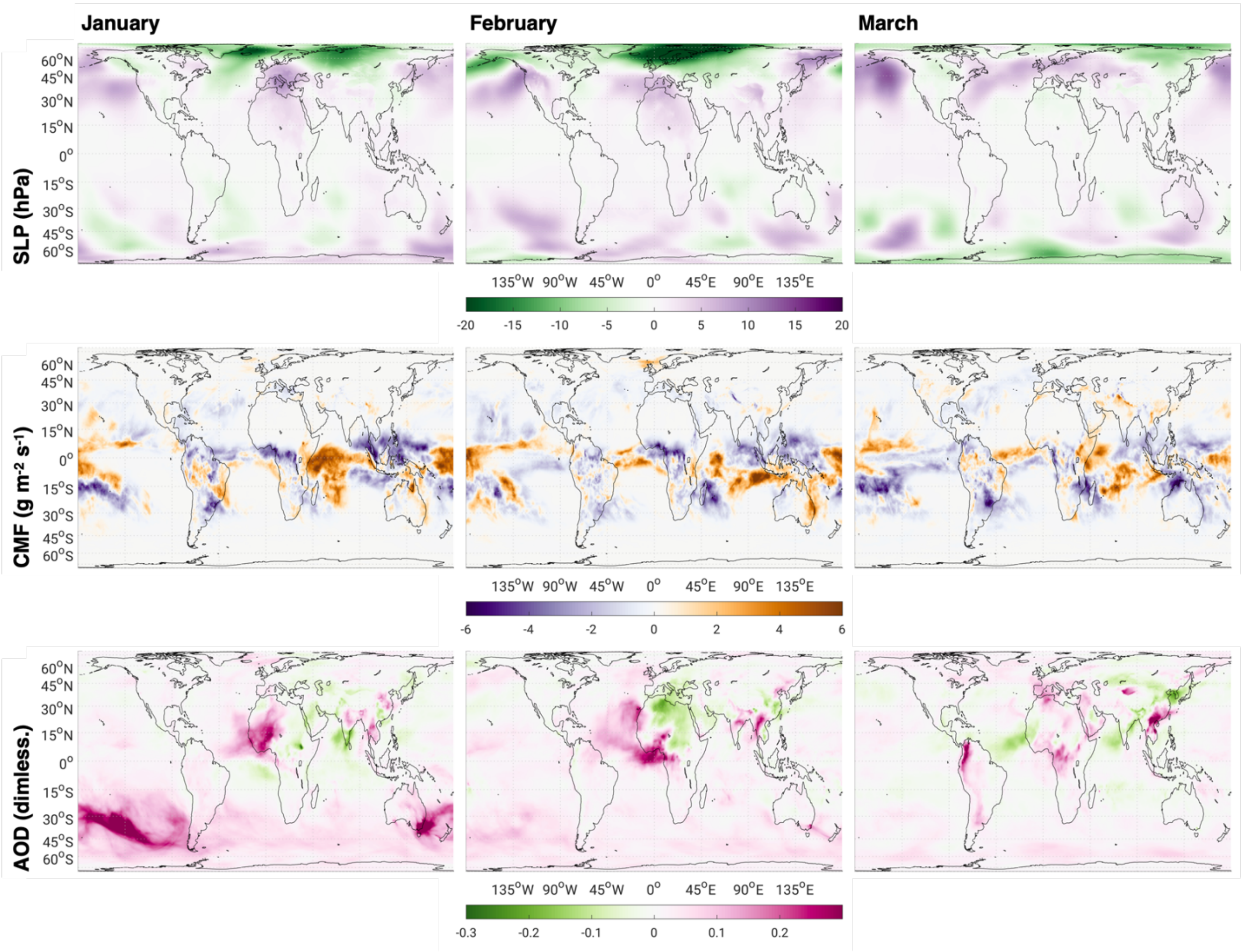

Fig. S10 (continued below …). Meteorological anomalies in 2020 for sea-level pressure (SLP; top), convective mass flux (CMF; middle), and aerosol optical depth (AOD) at 755 nm, the Oxygen A-band observed by OCO-2, (bottom) from MERRA-2. AOD anomalies, which can cause retrieval errors, are relatively small in the Northern Hemisphere during February–May 2020. The remaining plots are included for context.

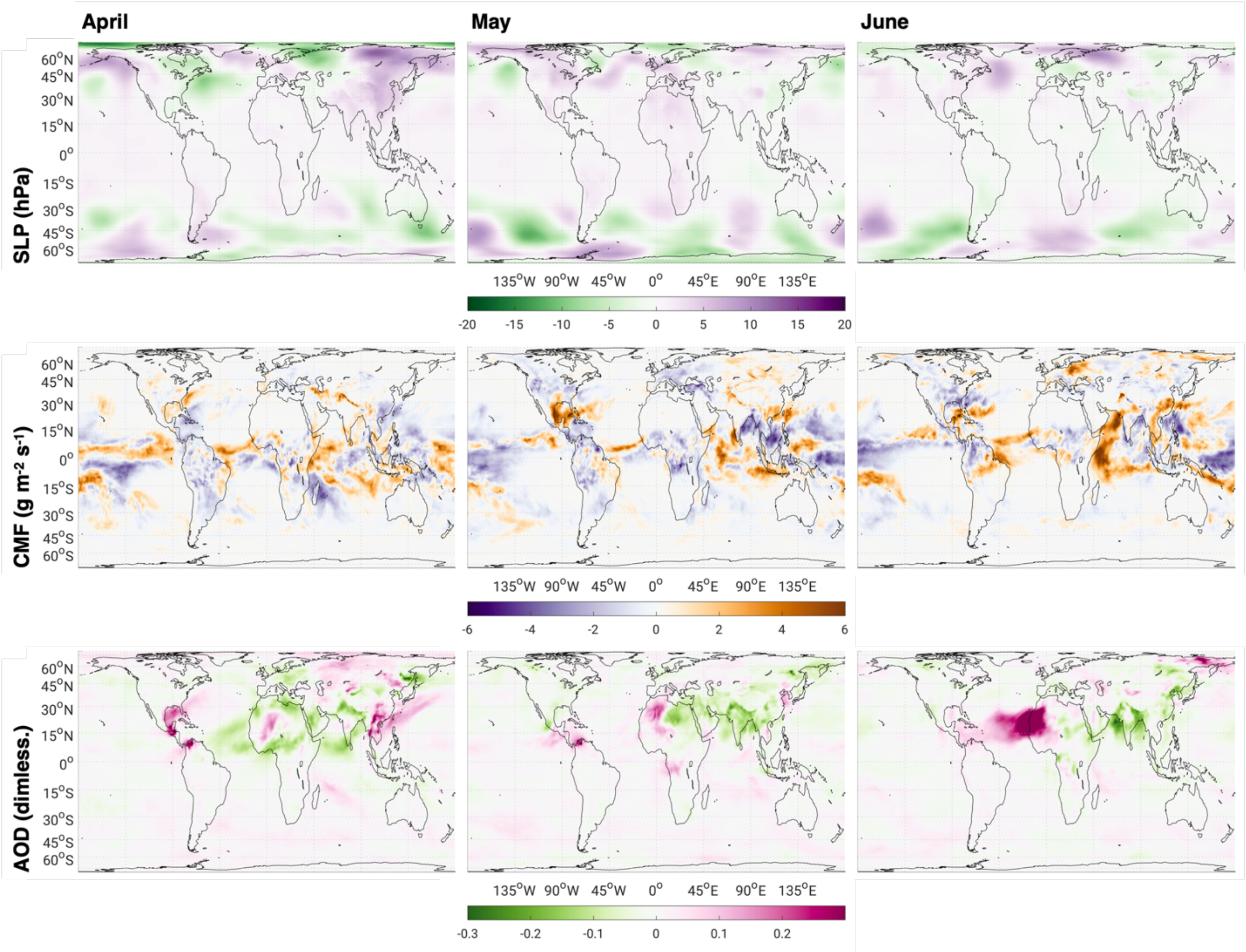

Fig S10 (... continued from above).

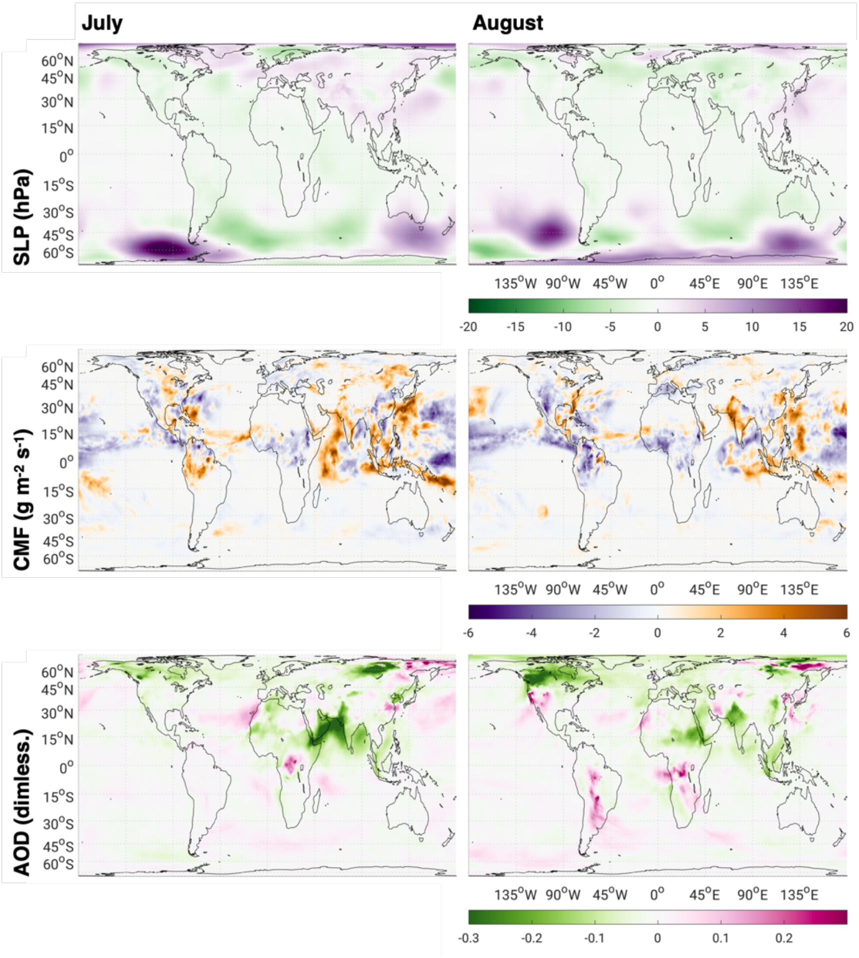

Fig S10 (... continued from above).

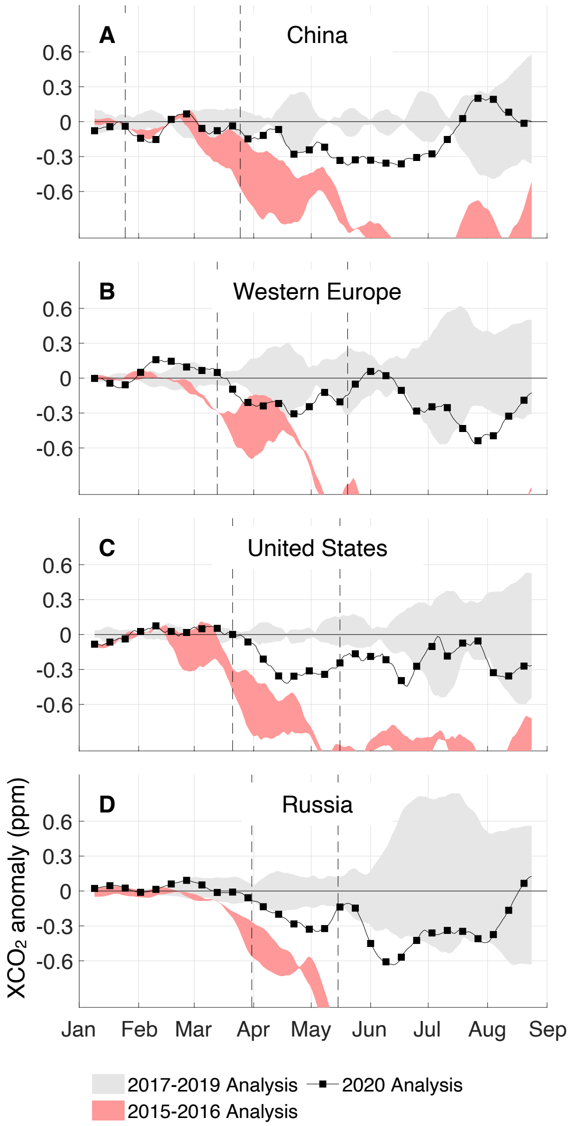

Fig. S11. Identical to Fig. 3, but with the range of the 2015–2016 ENSO years included. ENSO anomalies are negative because our surface fluxes underpredict the Northern Hemisphere land sink in these two years. Given the significant differences in the two ranges and the fact that ENSO conditions for 2020 were more reflective of 2017–2019, we exclude 2015–2016 from our analysis.

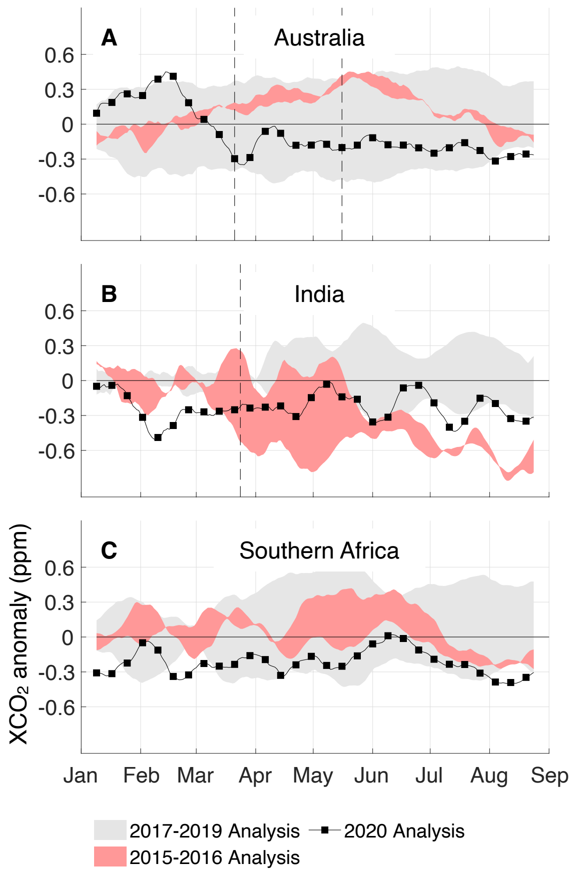

Fig. S12. Identical to Fig. 5, but with the range of the 2015–2016 ENSO years included.
